# Effects of Photobiomodulation Therapy on Oxidative Stress in Muscle Injury Animal Models: A Systematic Review

**DOI:** 10.1155/2017/5273403

**Published:** 2017-09-17

**Authors:** Solange Almeida dos Santos, Andrey Jorge Serra, Tatiane Garcia Stancker, Maíra Cecília Brandão Simões, Marcia Ataíze dos Santos Vieira, Ernesto Cesar Leal-Junior, Marko Prokic, Andrea Vasconsuelo, Simone Silva Santos, Paulo de Tarso Camillo de Carvalho

**Affiliations:** ^1^Postgraduate Program in Rehabilitation Sciences, Universidade Nove de Julho (UNINOVE), São Paulo, SP, Brazil; ^2^Postgraduate Program in Biophotonics, Universidade Nove de Julho (UNINOVE), São Paulo, SP, Brazil; ^3^Department of Physiology, Institute for Biological Research “Siniša Stanković”, University of Belgrade, Bulevar despota Stefana 142, 11060 Belgrade, Serbia; ^4^Department of Biology, Biochemistry and Pharmacy, Universidad Nacional del Sur, San Juan 670, 8000 Bahia Blanca, Argentina

## Abstract

This systematic review was performed to identify the role of photobiomodulation therapy on experimental muscle injury models linked to induce oxidative stress. EMBASE, PubMed, and CINAHL were searched for studies published from January 2006 to January 2016 in the areas of laser and oxidative stress. Any animal model using photobiomodulation therapy to modulate oxidative stress was included in analysis. Eight studies were selected from 68 original articles targeted on laser irradiation and oxidative stress. Articles were critically assessed by two independent raters with a structured tool for rating the research quality. Although the small number of studies limits conclusions, the current literature indicates that photobiomodulation therapy can be an effective short-term approach to reduce oxidative stress markers (e.g., thiobarbituric acid-reactive) and to increase antioxidant substances (e.g., catalase, glutathione peroxidase, and superoxide dismutase). However, there is a nonuniformity in the terminology used to describe the parameters and dose for low-level laser treatment.

## 1. Introduction

Muscle injuries are frequent in sports and workplace; more than 30% of the injuries seen in the physician's office are related to skeletal muscle. These injuries can occur through a variety of mechanisms, including those arising through direct trauma (e.g., laceration and contusion) and those through indirect trauma (e.g., ischemia, denervation, and strain), but the general process of muscle repair is similar in most cases [1]. After injury, the muscle repair process begins and is divided into interdependent phases: degeneration/ inflammation, regeneration, fibrosis/scar formation, and remodeling [2]. In addition, muscle damage causes an immediate acute ischemic response releasing reactive oxygen species (ROS) including superoxide anion, hydroxyl radical, and hydrogen peroxide. Moreover, ROS may also be released due to the migration, accumulation, and activation of polymorphonuclear cells. These events will finally provoke oxidation of cell membrane lipids, protein oxidation, proteolysis, and DNA fragmentation. Disruption of muscle structural integrity and function will induce changes in transport capacity, energy production, and ionic balance [3].

The oxidative stress has been reported to be involved in several diseases such as diabetes mellitus, neurodegenerative disorders (Parkinson's disease (PD), Alzheimer's disease (AD), and multiple sclerosis (MS)), cardiovascular diseases (atherosclerosis and hypertension), respiratory diseases (asthma), cataract development, and rheumatoid arthritis [4]. Many studies showed an increase ROS and oxidative damage markers in blood and tissues of humans and animals during and after muscle damage [1, 2, 5, 6]. After muscle injury, oxidative stress could be increased due to a number of potential sites for the ROS generation within the traumatized muscle.

Since the mid-1960s, the use of light energy as a therapy for inflammation and cell trophism has opened up a new research field to understand interaction between electromagnetic energy and biological tissue [7]. More recently, photobiomodulation therapy (PBMT) has been used to mitigate and delay muscle fatigue [8] in clinical [9, 10] and experimental [11] condition. There are studies showing that PBMT can improve mitochondrial function and mitigate ROS as well as reactive nitrogen species (RNS) generated during exercise training [12]. Thus, PBMT has been reported to modulate oxidative events, reducing oxidative stress in different situations [5, 13–15]. We performed this systematic review to identify animal research defining the effects of PBMT on experimental models of muscle injury and the impact of PBMT dosage.

## 2. Materials and Methods

### 2.1. Search Strategy

This search strategy was in accordance with the SYstematic Review Center for Laboratory animal Experimentation—SYRCLE guidelines for systemic review. For identification of studies included or considered for this review, from January 2006 to January 2016: EMBASE (Excerpta Medica Database), PubMed (Public/Publisher MEDLINE), and CINAHL (Cumulative Index to Nursing and Allied Health Literature). First, we selected key words from related articles. MeSH and SCOPUS international data lines were used to find more related key words with close meanings: (“oxidative stress”[MeSH Terms] OR (“oxidative”[All Fields] AND “stress”[All Fields]) OR “oxidative stress”[All Fields]) AND (“low-level light therapy”[MeSH Terms] OR (“low-level”[All Fields] AND “light”[All Fields] AND “therapy”[All Fields]) OR “low-level light therapy”[All Fields] OR “PBMT”[All Fields]) AND (“low-level light therapy”[MeSH Terms] OR (“low-level”[All Fields] AND “light”[All Fields] AND “therapy”[All Fields]) OR “low-level light therapy”[All Fields] OR (“low”[All Fields] AND “level”[All Fields] AND “laser”[All Fields] AND “therapy”[All Fields]) OR “low level laser therapy”[All Fields]) (“oxidative stress”[MeSH Terms] OR (“oxidative”[All Fields] AND “stress”[All Fields]) OR “oxidative stress”[All Fields]) AND (“phototherapy”[MeSH Terms] OR “phototherapy”[All Fields]) AND (“low-level light therapy”[MeSH Terms] OR (“low-level”[All Fields] AND “light”[All Fields] AND “therapy”[All Fields]) OR “low-level light therapy”[All Fields] OR (“photobiomodulation”[All Fields] AND “therapy”[All Fields]) OR “photobiomodulation therapy”[All Fields]) AND Photobiomodulation[All Fields].

**Figure 1:**
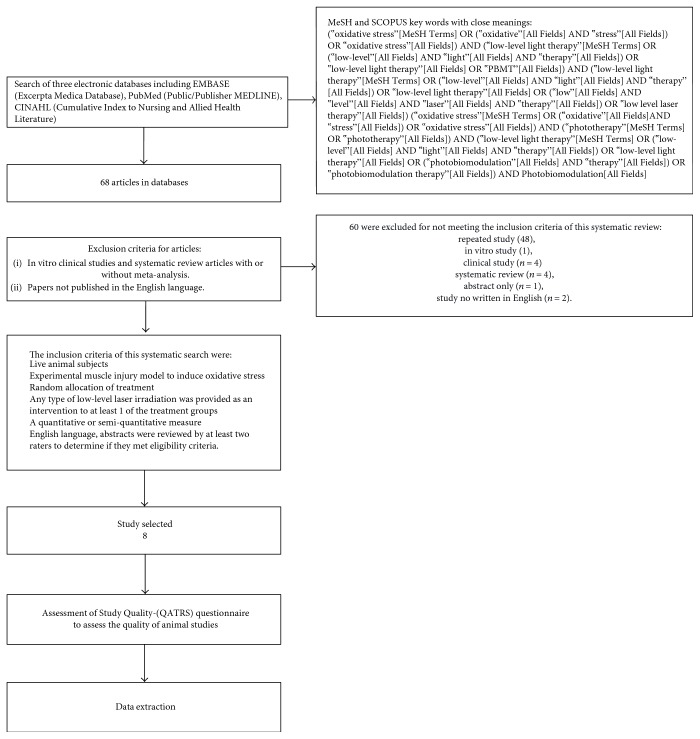
Flow diagram of the results of the study selection procedure.

The search was repeated following review of the eligible papers to specifically search for experimental methodologies and outcomes and parameters of photobiomodulation. We also reviewed the retrieved articles to identify possible additional studies (Figure 1).

### 2.2. Study Selection

We examined the title list and abstracts identified by the literature searches for potentially relevant studies. Two independent reviewers (SAS and AJS) applied a predetermined inclusion criterion to the full studies. Conflicts were resolved through a third independent researcher (PTC). The inclusion criteria of this systematic search were as follows:

1. Live animal subjects
2. Experimental muscle injury model to induce oxidative stress
3. Random allocation of treatment
4. Type of low-level laser irradiation was provided as an intervention to at least one of the treatment groups
5. A quantitative or semiquantitative measure
6. English language, abstracts were reviewed by at least two raters to determine if they met eligibility criteria.

Exclusion criteria:

1. *In vitro* clinical studies and systematic review articles with or without meta-analysis.
2. Papers not published in the English language.

### 2.3. Assessment of Study Quality

Potentially eligible articles were printed, reviewed, and critically appraised for quality rating by two independent reviewers. Systematic reviews are commonly performed in human research but rarely in animal research. Quality rating scales commonly used in human research may not be appropriate for animal studies, given that they do not consider issues like the appropriateness of the animal model being evaluated. For assessment of appropriateness, we used a quality scale developed by Tajali Bashardoust et al. [16]; this is a quality rating scale for an animal/tissue research scale (QATRS) questionnaire designed to assess the quality of animal studies. The QATRS is a 20-point scaled evaluation chart designed to assess randomization, blinding, similarity of the animal/tissue model with human applications, standardization and reliability of measurement techniques, management of study withdrawals, and appropriateness of statistical methods (Table 1).

## 3. Results

We found 68 articles in the databases. Abstracts were used to identify research that repeatedly appeared in more than 1 database (duplication of the same study) (*n* = 48). Thus, we prescreened 20 studies for full review. Among the 20 studies analyzed, 12 were excluded for not meeting the inclusion criteria of this systematic review: in vitro study (*n* = 1), clinical study (*n* = 4), systematic review (*n* = 4), abstract only (*n* = 1), and study not written in English (*n* = 2). We included 8 studies for critical evaluation of the effectiveness of PBMT in muscle injury, in which there are diverse treatment parameters of injuries were carried out.  Table 2 shows data extracted from the papers. The composition of samples from the 8 studies ranged from 18 to 90 animals, distributed randomly into 3–7 groups, with different studies presenting various primary outcomes; the most frequent oxidative stress biomarkers were catalase (CAT), superoxide dismutase (SOD), glutatione peroxidase (GPX), and biomarkers of lipid peroxidation (*n* = 4) (Table 3).

The studies used several models of experimental injury induction, and all of them were distributed in fatigue [5, 15], cryoinjury [2, 6], traumatic injury [1, 3], and in lesser occurrence Carrageenan [17] and adrenaline [14]. Six studies used male animals and two used female animals. The studies were analyzed by a range of methodological rigor called the QATRS encompassing various aspects that enable better quality control of the experimental studies. Study scores ranged from 17 to 19 points on a scale of 0–20 (Tables 4 and 1). When analysis of the positive effects was statistically significant, eight studies found positive effects (Table 5).

## 4. Discussion

In this review, articles focusing primarily on the effects of PBMT on oxidative stress in experimentally muscle injury were analyzed; for all articles, there was no unanimity regarding the outcome measures, nor the methods used to measure these outcomes. Frequently, different classifications and evaluations were used to designate similar variables. This may be due to the multifactorial etiology of the disease and the fact that its pathogenesis is still unknown [6]. Enwemeka et al. [18] stated that such failures are the causes of inconsistencies in the literature, especially with PBMT.

The most frequently analyzed variables were histology, creatine kinase, CAT, SOD, GPX, oxide nitric production, and TBARS. Based on the outcomes listed (Table 3), positive outcomes depend on the proper use of two key factors: an experimental model that mimics muscle injury and the use of the intervention employed.

Possibly LLLT and LEDT improves mitochondrial function, O_2_^•−^ dismutation via SOD and decreases formation of ONOO^−^. In addition, LLLT can reduce H_2_O_2_ via CAT and GPX and can reduce the formation of hydroxyl radicals, which contribute to lower muscle cell membrane damage, as evidenced by a lower lipid peroxidation [19]. The reviewed studies had focused on the analysis of only one muscle, and 60% of these investigated the alterations suffered in the gastrocnemius muscle [1, 3, 5, 17] and the induction medium was distributed in 25% fatigue [5], traumatic lesion 25% [1, 3], and in lesser occurrence Carrageenan [17] and adrenaline [14] with 12.25% (Table 2).

According to Assis et al. [6], the inflammatory phase of the muscle injury is accompanied by an increased ROS and RNS production and a reduced activity in antioxidant enzymes. This imbalance between prooxidants and antioxidants, in favor of prooxidants, can generate oxidative and nitrative stress in the tissue that contributes to activate NF-*κ*B, a pleiotropic transcription factor responsible for multiple changes in gene expression in the inflammatory process.

The muscle traumatic injuries especially in the acute phase benefited from the ROS, which in combination with growth factors and cytokines, are important to the muscle repair due to the redirection of myogenic precursor cells (satellite cells to the injury site). Cause apoptosis in satellite cells [20] as differentiated adult skeletal muscle fibers has scarce ability to repair and regenerate themselves when a cellular injury exists; satellite cells have the capacity to proliferate and differentiate, with vital properties to repair the injured tissue [21]. In this context, satellite cells and their response to oxidative stress are important to mature skeletal muscle performance. In addition, photobiomodulation with low-level laser caused a protective effect on myoblasts [22].

However, high levels of ROS for a long period in the injured area can cause oxidative harm (secondary damage) by directly reaching vital cell constituents, such as lipids, proteins, and DNA, in addition to interfering negatively in the differentiation of muscle cells [2].

Therefore, we can verify that both the traumatic lesions induced by the use of cold are adequate as models of ROS generation and consequently oxidative stress. The literature has also demonstrated the use of exercise of high intensity [15] with the aim of generating muscle fatigue and consequently oxidative stress can be good indicators for this type of analysis.

Although the focus of the current review is centered on the parameters of dosimetry used during photobiomodulation, it aims to mitigate the oxidative stress and improve the antioxidant ability of the skeletal muscle. In this respect, we realized that there is an agreement on the type of wavelength used in studies ranging from red (632,8 nm) [14, 17] to the infrared (780, 808, and 904 nm) [1–3, 5, 6, 14, 17], being that 30% of the studies offered to make a comparison between the wavelengths (780/660 nm) [2] and (632.8/904 nm) [14, 17], being that in three comparisons these studies obtained better results in the use of infrared (Table 4).

The effective tissue penetration of light and specific wavelength of light absorbed by photoacceptors are two of the major parameters to be considered in light therapy. In the tissue, there is an “optical window” that runs approximately from 650 nm to 1200 nm where the effective tissue penetration of light is maximized [15].

Regarding the power of light used in the studies, we also observed a wide variation between 5 mW and 100 mW, being that 60% of these studies ranged between 35 and 45 mW. Regarding the energy densities (fluence), 100% of the studies described the dose that ranged between 180 and 5 Jcm^2^. If on the one hand, all the studies analyzed described the parameters mentioned above, on the other hand 50% did not describe what area of the laser beam [1, 2, 14, 17] was used, as well as power density (irradiance) 30% [1, 14, 17] and energy in Joules 50% [1, 14, 17] (Table 4). The absence of these parameters weakens the studies once the literature has shown that the results of photobiomodulation depends on the irradiation time and dose used. If we take into account that different areas of beam and powers propose different irradiation times and densities of different energies, the reproducibility of these studies are threatened. This can be verified at the great variations presented in the irradiation time per point.

The fluence (energy density) used is generally between 1 and 20 J/cm^2^ while the irradiance (power density) can vary widely depending on the actual light source and spot size; values from 5 to 50 mW/cm^2^ are common for stimulation and healing, while much higher irradiances (up to W/cm^2^) can be used for nerve inhibition and pain relief. PBMT is typically used to promote tissue regeneration, reduce swelling and inflammation, and relieve pain and is often applied to the injury for 30 seconds to a few minutes or so, a few times a week for several weeks [23]. According to Araruna Alves et al. [24], all these aspects must be disclosed in scientific research so that the study becomes reproducible and has measurable outcomes. Therefore, with standardization of the use of the laser, its mechanism of action and its results would be clarified, thus ensuring positive results with the use of photobiomodulation and advances in rehabilitation sciences.

**Figure 2:**
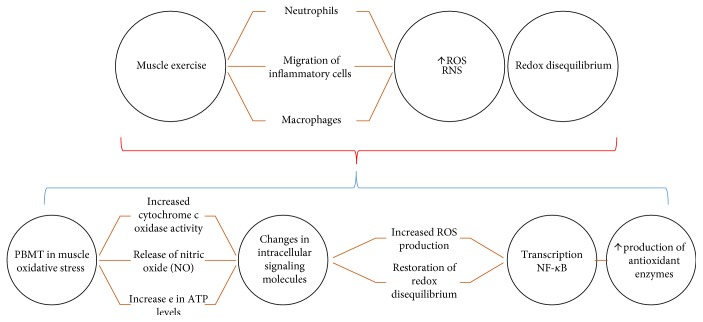
Schematic representation of mechanisms of photobiomodulation- PBMT action on muscle oxidative stress—the oxidative stress generated during exercise or injury is linked to the migration of cells of inflammatory cells (such as neutrophils and macrophages) to the source of ROS. The increase of the reactive oxygen species triggers a redox state de-balancing. Basic biological mechanism behind the effects of PBMT: red and infrared light is absorbed by cytochrome c oxidase (IV complex of the mitochondrial respiratory chain). PBMT triggers increased ROS production, such as superoxide (O_2_) and hydrogen peroxide (H_2_O_2_), leading to the restoration of redox imbalance because of higher production of antioxidant enzymes. Altering the redox state in the cells induces the activation of intracellular signaling, increasing the activation of the transcription factor redox sensitive.

It is accepted that the migration of inflammatory cells (such as neutrophils and macrophages) to the muscle site required during exercise and under this condition cells of inflammatory cells are a source of ROS. Thus, it is possible to imagine that the oxidative muscular homeostasis linked to PBMT could be mediated by its anti-inflammatory action, inhibiting/attenuating the of inflammatory cell migration, then, the ROS source. In addition, PBMT application has been reported to induce superoxide dismutase (SOD) increases, in which could contribute to alleviate the muscle damage by reducing oxidative stress. In fact, SOD is an enzyme with elevated capacity of scavenging O_2_ radicals. It has also been shown that some light wavelengths are absorbed by hemoglobin, releasing nitric oxide from the nitrosothiols in the beta chain of the hemoglobin molecule (Mittermayr et al. [25]; Vladimirov et al. [26]; and Vladimirov et al. [27]. Since during exercise (mainly aerobic) there is a greater influx of blood to the active muscle, LLLT could potentialize the release of nitric oxide to modulate oxidative stress (Figure 2).

Based on the results of the studies included in this review, there is sufficient evidence to suggest that photobiomodulation is an effective short-term approach for reducing TBARS levels and antioxidants levels (Table 5). Furthermore, the parameters used for PBMT in the studies examined, such as laser output, irradiation distance, irradiation frequency per day, number of treatment sessions, irradiated energy per day, and the total energy irradiated, did not meet the current recommendations for reproducible studies. It is necessary to establish the optimal dosage and exposure levels necessary for achieving results in decreased oxidative stress in muscle injury.

## 5. Conclusions

Although the small number of studies limits the systematic review on photobiomodulation, evidence was found to suggest que PBMT is an effective short-term approach for reducing oxidative stress in muscle injury. However, lack of uniformity in the terminology used to describe parameters and the dose used for PBMT limits the ability to reach firm conclusions.

## Figures and Tables

**Table 1:** Representation of the quality rating scale items for animal/tissue research scale (QATRS).

Item	Rating		
	Yes (2)	Partial (1)	No (0)
(1) Animals/tissue samples were randomly allocated to groups.			
(2) The animals/tissue samples were similar across comparison groups.
(3) The tissue/animal model study was appropriate for the biological properties/questions being evaluated.
(4) The animal model used was appropriate to make inferences in terms of human application? (tissue similar to, or is human tissue).
(5) Objective measurements were performed using sufficient standardization of measurement techniques and appropriate instrumentation.
(6) Reliability of measurements was reported or referenced to indicate sufficient consistency of the outcomes analyzed.
(7) Are all animals entered into the study accounted for? (All were analyzed or reasons for withdrawal were noted).
(8) 90% of the animals entered were included in the data analysis.
(9) The between group/time statistical comparisons used appropriate statistical methods.
(10) Measures of variability and confidence intervals were provided to indicate the range/size of the effects observed.
Total score (/20)

**Table 2:** Study characteristics of selected experimental controlled animal studies on low-level laser irradiation effects on oxidative stress.

Authors	Animal type	Gender	Animal race	Age (months)	Weight (g)	Induction model	Site injury	QATRS
Guaraldo et al. [5]	Rat	Male	Wistar	24	517.7 ± 27.54	Fatigue	Gastrocnemius	17
Ribeiro et al. [2]	Rat	Male	Wistar	—	250 ± 15	Cryolesion	Tibialis anterior	19
Oliveira Silva et al. [15]	Mice		Mdx/C57 BL	4	—	Fatigue	Gastrocnemius/Soleus	19
Silveira et al. [3]	Rat	Male	Wistar	Adult	250–300	Trauma	Gastrocnemius	19
Assis et al. [6]	Rat	Male	Wistar	Adult	300	Cryolesion	Tibialis anterior	19
Davila et al. [17]	Rat	Female	Wistar	5	200 ± 20	Carrageenan *λ* (type IV)	Gastrocnemius	19
Servetto et al. [14]	Rat	Female	—	—	250–300	Adrenaline	Left posterior limb muscle	19
Rizzi et al. [1]	Rat	Male	Wistar	—	250–300	Impact blunt trauma	Gastrocnemius	19

**Table 3:** Study characteristics of selected experimental controlled animal studies on low-level laser irradiation effects on oxidative stress.

Authors	Sample size	Group number	Number of animals/group	Dependent variables
Guaraldo et al. [5]	30	05	06	Biomarkers of oxidative stress (CAT, SOD, and GPX); biomarkers of lipid peroxidation.
Ribeiro et al. [2]	80	06	05/15	Chemoluminescence; protein oxidation; antioxidant enzyme activity
Oliveira Silva et al. [15]	28	04	07	Histology; quantification total creatine kinase; protein carbonyl; detection of superoxide dismutase
Silveira et al. [3]	18	03	06	Serum creatine kinase activity; hydroxyproline measurement; superoxide anion production; lipid peroxidation assay; superoxide dismutase; protein determination
Assis et al. [6]	60	03	20	Muscle evaluation; muscle morphological analysis; lipid peroxidation; NO production; immunoblotting; dot blot (for detection of nitrotyrosine formation); cytokine measurements (ELISA); total RNA isolation and real-time PCR
Davila et al. [17]	70	07	10	Histological analysis; plasma collection; muscle tissue collection
Servetto et al. [14]	48	06	08	Plasma collection; muscle tissue collection; spectrophotometry in plasma
Rizzi et al. [1]	90	3	30	Histology; collagen quantification; TBARS analysis; Western blot analysis; electrophoretic mobility shift assay

**Table 4:** Study characteristics of selected experimental controlled animal studies on low-level laser irradiation effects on oxidative stress.

Authors	Wavelength (nm)	Energy density (J/cm^2^)	Energy (J)	Power density (W or mW/cm^2^)	Spot size (cm^2^)	Irradiation time per point (sec)	Duration of treatments (days)	Treatment frequency (days)	Laser frequency (Hz)	Power (mW or W)
Guaraldo et al. [5]	808	144	4	1.071	0.028	40	—	6 weeks	—	100 W
Ribeiro et al. [2]	780/660	10	3.2	1	—	10	7	1, 3, and 7 after the induction of injury	—	40 mW
Oliveira Silva et al. [15]	808	107	—	1027	0,028	100	3	Consecutive days	—	30 mW
Silveira et al. [3]	904	5	2.5	400	0.10	12.5	5	2, 12, 24, 48, 72, 96, and 120 hours after the trauma	9.500	40 mW (peak power 70 W)
Assis et al. [6]	808	180	1.4	3.8	0.00785	47	4	Consecutive days	—	30 mW
Davila et al. [17]	632.8/904	9.5	—	—	—	60/47	10	Consecutive days	—	5/12 mW
Servetto et al. [14]	632.8/904	9.5	—	—	—	60/47	7	Consecutive days	—	5/12 mW
Rizzi et al. [1]	904	5 J	—	—	—	35	7 or 14	Daily	—	45 mW

**Table 5:** Study characteristics of selected experimental controlled animal studies on low-level laser irradiation effects on oxidative stress.

Authors	Positive effects: statistically significant	Positive effects: not significant	No effect
Guaraldo et al. [5]		X	
Ribeiro et al. [2]		X	
Oliveira Silva et al. [15]	X		
Silveira et al. [3]	X		
Assis et al. [6]		X	
Davila et al. [17]		X	
Servetto et al. [14]	X		
Rizzi et al. [1]		X	
